# Kinematic analysis of sensorimotor control during the craniocervical flexion movement in patients with neck pain and asymptomatic individuals: a cross-sectional study

**DOI:** 10.1186/s12984-023-01133-8

**Published:** 2023-01-17

**Authors:** Elena Bocos-Corredor, Filippo Moggioli, Tomás Pérez-Fernández, Susan Armijo-Olivo, Cristina Sánchez, Juan Nicolás Cuenca-Zaldívar, Josué Fernández-Carnero, Aitor Martín-Pintado-Zugasti

**Affiliations:** 1 Departamento de Fisioterapia, Facultad de Medicina, Universidad San Pablo-CEU, CEU Universities, Madrid, Spain; 2 Faculty of Business and Social Sciences, University of Applied Sciences, Caprivistr. 30A, 49076 Osnabrück, Germany; 3 Department of Physical Therapy, Faculty of Rehabilitation Medicine, University of Alberta, 3-48 Corbett Hall, Edmonton, AB Canada; 4 Department of Physical Therapy, Occupational Therapy, Rehabilitation and Physical Medicine, Rey Juan Carlos University, Madrid, Spain; 5 La Paz Hospital Institute for Health Research, IdiPAZ, Madrid, Spain; 6 Grupo de investigación en Neurociencia cognitiva, dolor y rehabilitación en ciencias de la salud, Universidad Rey Juan Carlos, NECODOR, Madrid, Spain; 7 Departmento de Ingeniería de Sistemas de Información, Universidad San Pablo-CEU, CEU Universities, Madrid, Spain; 8 Universidad de Alcalá, Facultad de Medicina y Ciencias de la Salud, Departamento de Enfermería y Fisioterapia, Grupo de Investigación en Fisioterapia y Dolor, 28801 Alcalá de Henares, Spain; 9 Research Group in Nursing and Health Care, Puerta de Hierro Health Research Institute-Segovia de Arana (IDIPHISA), Madrid, Spain; 10 Primary Health Center, “El Abajón”, Las Rozas de Madrid, Madrid, Spain

**Keywords:** Movement disorders, Neck muscles, Exercise, Headache, Kinematics, Biomechanical phenomena

## Abstract

**Background:**

Patients with craniocervical pain have shown reduced performance in the craniocervical flexion test (CCFT). However, there is limited evidence of other possible kinematic alterations not assessed in the context of the CCFT. Previous studies on other functional or planar movements have reported alterations in sensorimotor control (e.g., range of motion [ROM], velocity, or smoothness) in subjects with neck pain. The objective of this study was to explore the association between sensorimotor control variables associated with craniocervical flexion movement and different characteristics related to pain, age, disability, and fear of movement in individuals with non-traumatic chronic neck pain and asymptomatic controls.

**Methods:**

This was an observational, cross-sectional study in patients with non-traumatic neck pain and asymptomatic participants. Regression models were used to assess whether descriptive characteristics of the sample, including: (a) age, (b) intensity of pain, (c) neck disability, (d) chronicity of pain, and (e) fear of movement could explain sensorimotor control variables such as ROM, velocity, jerk, head repositioning accuracy, and conjunct motion. All these variables were recorded by means of light inertial measurement unit sensors during the performance of three maximal repetitions of full range craniocervical flexion in the supine position.

**Results:**

A total of 211 individuals were screened and 192 participants finished the protocol and were included in the analyses. Participants had an average age of 34.55 ± 13.93 years and included 124 patients with non-traumatic neck pain and 68 asymptomatic subjects. Kinesiophobia partially explained lower craniocervical flexion ROM (p = .01) and lower peak velocity in flexion (P < .001). Age partially explained increased craniocervical extension ROM (P < .001) and lower peak velocity in flexion (P = .03). Chronicity partially explained increased lateral flexion conjunct motion (P = .008). All models showed low values of explained variance (< 32%) and low absolute values of regression coefficients.

**Conclusions:**

This study did not find a clear relationship between population characteristics and sensorimotor control variables associated with the craniocervical flexion movement. Kinesiophobia might have some association with reduced ROM in craniocervical flexion, but further research in this field is needed in large samples of patients with higher levels of kinesiophobia pain or disability.

## Background

Neck pain is a very common musculoskeletal disorder and a major public health burden with high prevalence, incidence, and years lived with disability worldwide [1]. The annual prevalence of neck pain ranges from 15–75.1% [2] and has been estimated to be as high as 71% of the adult population having at least one episode of neck pain in their lifetime [3]. This represents a major public health problem associated with high socio-economic costs in terms of absenteeism from work and medical expenses [4]. Between 50 and 75% of people who have suffered from neck pain continue to experience symptoms 1–5 years after the onset of symptoms or suffer recurrent episodes [5].

Previous studies have related the onset and perpetuation of craniocervical pain to multiple alterations of the cervical sensorimotor system compared to asymptomatic population, such as reduced muscular strength and endurance of the cervical muscles [6–8], altered proprioception [9, 10], impaired kinematics [11, 12], or changes in muscle morphology [13]. Decreased active range of motion (ROM), impaired movement accuracy, impaired repositioning accuracy, reduced speed of movement, or decreased smoothness of neck movement have also been observed [14–18].

The craniocervical flexion test (CCFT) specifically assesses the function of the deep neck muscles of the cervical region. [10, 19, 20]. It consists of a controlled upper cervical flexion action performed during five incremental stages of pressure, increasing the range of motion of craniocervical flexion in the supine position [20]. Correct performance of the test implies the ability to achieve and maintain an isometric contraction in each of the incrementing stages without compensatory movements such as retraction, lift of the head off the table, or overuse of the superficial cervical flexors [20]. Patients with craniocervical pain have shown reduced performance in the CCFT compared to asymptomatic individuals [21–24]. Therefore, the performance of the deep flexor muscles is frequently considered in the evaluation of patients with neck pain [25, 26], as well as in the prescription of specific therapeutic exercise programs [19].

Although CCFT has been frequently investigated in patients with craniocervical pain, it is unclear whether this specific craniocervical flexion movement might be altered in relation to other parameters (e.g., speed, smoothness. or joint position error) than those currently obtained from the CCFT, as well as the relevance of measuring these parameters. Several studies have evaluated the aspects of sensorimotor control in multiple cervical movements rather than during craniocervical flexion [16–18]. However, to our knowledge, the kinematics of the craniocervical flexion movement specifically have not been explored by assessing variables of sensorimotor control, such as speed or smoothness, by comparing subjects with non-traumatic neck pain with asymptomatic controls. Moreover, it is unclear whether other factors of the studied population different than pain, such as disability, chronicity, age or fear of motion could influence the performance of this movement. Previous research has observed alterations in the performance of the CCFT in elderly subjects when compared to young participants [27]. Also, a recent study of Devecchi et al. have highlighted the association between fear of pain and neuromuscular and kinematic adaptations observed in people with a history of neck pain [28]. Previous research specifically investigated the ROM of the craniocervical flexion, and observed that patients with neck pain or headache had reduced ROM when compared with asymptomatic subjects during full range movement [29] or during the stages of CFFT [21]. Ernst et al. [29] emphasized the need for a separate ROM assessment of the upper cervical spine and recommended examining the capability of upper craniocervical ROM to discriminate between healthy subjects and symptomatic patients in future studies.

It is possible that kinematic analysis of sensorimotor control of this specific movement may provide relevant information for understanding the mechanisms underlying craniocervical pain or assessing the effects of therapies in relation to changes in patients´ sensorimotor control. Moreover, the kinematics of craniocervical flexion assessed as an independent movement could potentially be used as a alternative form of assessment of the deep cervical flexors, since the limited reliability of the CCFT has recently considered to affect its suitability [30]

Therefore, this study aimed to explore whether different characteristics related to pain intensity, age, neck-pain related disability, and fear of movement explain sensorimotor control performance (ROM, velocity, jerk, head repositioning accuracy, and conjunct motion) during the craniocervical flexion movement on a sample of non-traumatic chronic neck pain patients and asymptomatic controls.

## Methods

### Design

This was a descriptive, observational, cross-sectional study, including patients with non-traumatic neck pain and a control group of asymptomatic participants based on a previously described study protocol [31].

This study was designed and the findings are reported in accordance with the Strengthening the Reporting of Observational Studies in Epidemiology (STROBE) [32].

The study was approved by the Research Ethics Committee of CEU San Pablo University (495/21/39). Participants provided informed written consent before being enrolled into the study and they were able to withdraw their consent at any time during the study, in compliance with the WHO standards and the Declaration of Helsinki [33].

### Sample and selection

The sample was composed of a group of patients with non-traumatic subacute and chronic neck pain and another group of asymptomatic subjects. We recruited a non-probabilistic convenience sample via flyers, online forms through social networks, e-mail, or direct verbal communication at San Pablo-CEU University, the CEU San Pablo University clinic, as well as in private physiotherapy clinics in the Community of Madrid.

Patients with neck pain were eligible to be included in the study if they were 18–65 years old and fulfill the following selection criteria: (a) neck pain of at least 1 month of evolution, (b) neck pain from non-specific mechanical origin associated or not with primary headache (e.g. migraine or tension-type headache), shoulder pain, or upper limb pain. Patients with neck pain were excluded if they presented any of the following criteria: (a) complex regional syndrome, (b) previous surgeries in the neck and/or head region, (c) vestibular alterations, (d) otogenic or idiopathic vertigo/dizziness, (e) presence of tumors in the craniocervical region, (f) previous fracture in the head or neck region, (g) osseous deformities in the thoracic, cervical, or cranial region.

Asymptomatic subjects should not have any pain in the cervical region during the last year and no previous treatment for neck pain. The exclusion criteria were the same as described for patients with neck pain.

Once deemed eligible, subjects were asked to read and sign the informed consent prior to participation and then were invited to participate in the study, consisting of one session in which all variables described below were assessed.

### Instrumentation and measures

Prior to sensorimotor control testing, subject demographic characteristics such as age, gender, weight, height, and dominant side were recorded. In addition, the following pain or descriptive variables were collected for all participants: (a) mean intensity of pain during the last month (visual analog scale), (b) neck disability measured using the Spanish version of the Neck Disability Index (NDI) [34], (c) duration of pain since pain started (months). Participants were considered asymptomatic in case these three variables were recorded with a score of 0. Then, these variables were included in the regression statistical analysis described below as continuous variables.

### Primary outcomes: sensorimotor control assessment

Sensorimotor control kinematic variables were recorded by means of small (4 cm × 4 cm × 8 cm), light (< 200 g) Inertial Measurement Unit (IMU) sensors (Werium Solutions©, Madrid, Spain), which integrate a 3D accelerometer, a gyroscope, and a magnetometer. This inertial sensor technology has previously shown good to excellent intra-rater and inter-rater reliability in the measurement of general cervical ROM (ICC = 0.93) [35] and craniocervical flexion (ICC > 0.80) [36].

An independent assessor administered testing procedures, blinded to subject group status (neck pain or asymptomatic). Another researcher recorded the demographic variables and was aware of the subject’s pain status. As reported by similar previous research [37, 38] performance and detection biases during this type of testing procedure are less likely, since these procedures use automatic computerized data collection and processing.

**Fig. 1:**
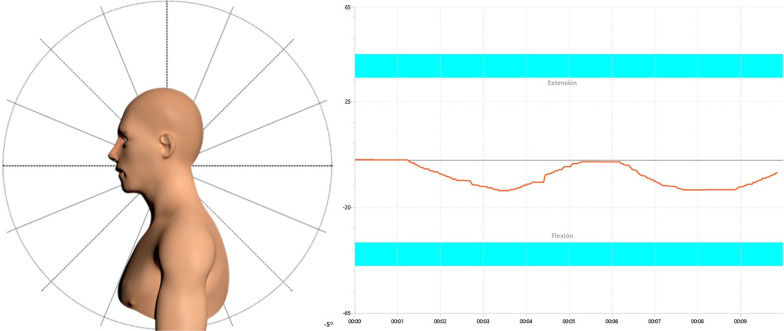
Displayed computer biofeedback screen for real-time monitoring of craniocervical flexion movement

The kinematics of the craniocervical flexion movement in supine position were evaluated for each subject enrolled in the study. Participants received instructions on testing procedures (see below). During the test, the assessor was able to monitor on real time the values of ROM displayed on a computer screen (Fig. 1).

Before starting with the test, participants were asked to sit naturally in a standard chair with the feet well supported on the floor and the neck and head in a neutral comfortable position with their hands resting on their thighs. Patients performed three repetitions of flexion/extension, lateral inclination, and rotations (to both sides), serving as a warm-up exercise before the measurement of craniocervical flexion.

Then, the assessor asked the subjects whether they had any questions before starting the test. Then subjects were asked to perform three consecutive active movements of the craniocervical flexion movement in supine position as described in the section below.

### Secondary outcomes

All participants completed a questionnaire related to fear of movement, which is also described below. The Spanish version of the Tampa Scale for Kinesiophobia (TSK11) [42] was used to assess fear of movement and injury. This self-reporting questionnaire includes 11 items that are rated on a four-point scale, where 4 represents “strongly agree” with the statement and 1 represents “strongly disagree”. Scores range from 11 to 44, where higher scores indicate higher fear of movement. This Spanish version showed good reliability and validity, with an internal consistency of α = 0.79.

### Craniocervical flexion in supine position

**Fig. 2:**
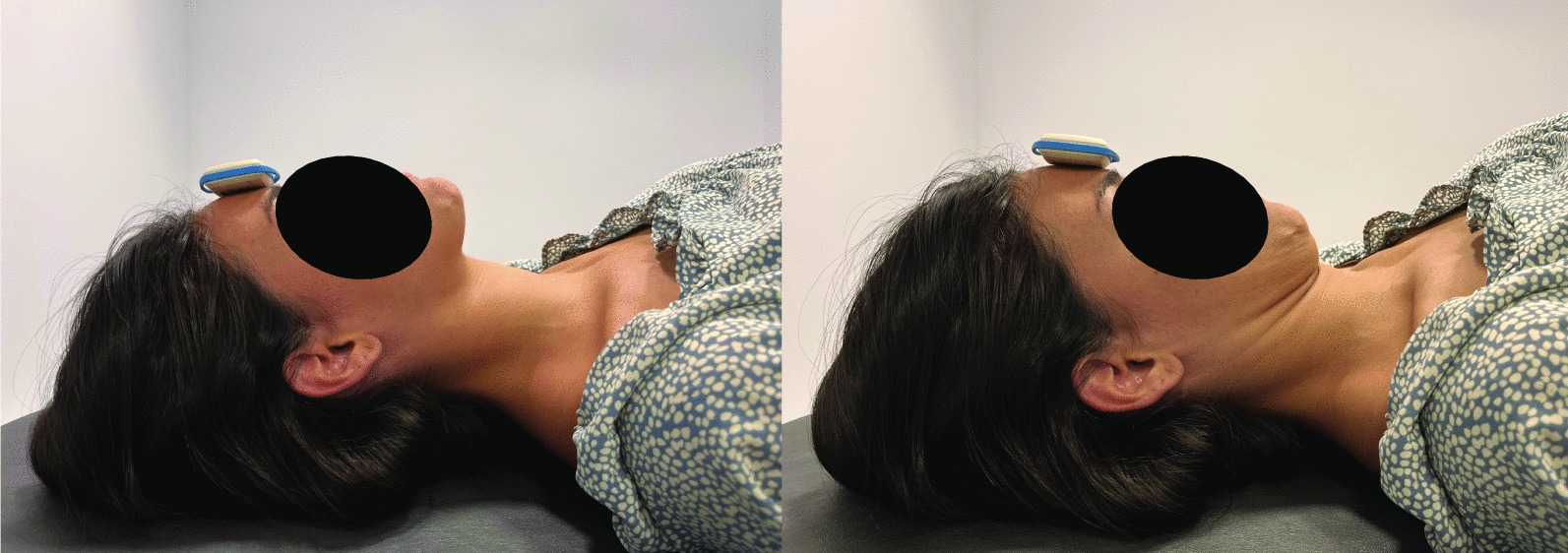
**a** Starting neutral position and inertial sensor placement. **b** Full-range craniocervical flexion and inertial sensor placement

One wireless wearable sensor was adhered to the center of the forehead (defined as the place where the lines that bisect the forehead longitudinally and horizontally cross in supine position) before starting the test (Fig. 2a).

Placement of the sensor with these landmarks has shown to be a reliable method for measuring craniocervical flexion ROM in previous research [36]. Participants were placed in a relaxed supine position with the forearms resting on the abdomen, the knees flexed, and the neck in a neutral comfortable position. The assessor visually assessed that the cranio-cervical spine was in a mid-position in which the subjects’ chin and forehead were horizontal and an imaginary line, which extended from the tragus of the ear to bisect the neck longitudinally, was parallel to the plinth[39]. Subjects were reminded to stay and memorize the starting neutral position of the head (Fig. 2a) and return to this position as accurately as possible after each of the three repetitions. Once the participant was in this position, the sensor was set and calibrated to the starting position. At this moment, patients were asked to perform three repetitions of the full-range craniocervical flexion movement (Fig. 2b), consisting of an anterior rotation of the head in a nodding action, feeling the back of their heads sliding up on the table. During the test, the assessor provided standardized verbal cues to guide the process with a correct technique if necessary.

Additionally, the movement was repeated in case the assessor detected signs of compensation, such as lower cervical flexion, neck retraction, or overuse of the superficial flexor muscles.

This procedure was verbally explained to participants through the following standardized instructions to ensure same information was explained to all subjects: “Please lie on your back with your knees bent and your feet resting on the table. The starting position will be relaxed with the gaze directed vertically towards the ceiling. Then, please perform an anterior rotation of the head in a controlled manner without moving your neck, such that the head rotates slightly, reaching as far as possible. The posterior side of the head will slide smoothly on the table during the movement and the head should not separate from the table or push into the table during the movement. You must perform this movement three times, returning to the starting position after each one and holding the position of maximum flexion for three seconds in each repetition”.

### Sensorimotor control data processing

A software application computed and exported the complete ROM of the participants, expressed as angles from the calibrating starting position in the three axes, sampled every 20 ms. In addition, data smoothing filters were applied to avoid peaks and variations not corresponding to the trend of each data set. The analysis of these data allowed for the calculation of the following variables during three repetitions of craniocervical flexion:

- Active craniocervical flexion ROM expressed as the maximal angular displacement (°) achieved in any of the three repetitions
- Active craniocervical extension ROM when trying to return to the neutral position from the craniocervical flexion motion, also expressed as the maximal angular displacement (°). This variable represents poorer accuracy when trying to achieve the neutral position. It is different from the variable “Head repositioning accuracy”, since this excessive extension motion was frequently observed immediately before the patient tried to further change the position to come to the point in which they were supposed to have achieved the real neutral position between repetitions.
- Peak velocity in craniocervical flexion and extension independently, expressed as the maximal angular velocity (°/s): calculated as the discrete derivative of angular orientation applying a standard smooth filtering algorithm.
- Smoothness of motion expressed as maximal movement jerk peak (º/s^3^): calculated as the third discrete derivative of the angular orientation (change in acceleration) [40, 41].
- Head repositioning accuracy expressed as angular displacement (°): calculated as the absolute repositioning error considering the maximal difference between the neutral starting position (set up at the beginning of each movement) and the positions reached when the patient tried to come back again to the neutral after returning from flexion. The maximal difference observed during all the three repetitions was used for the analysis.
- Peak conjunct motion (°): calculated as the angular displacement occurring in a different anatomical plane to the one that is being tested (movement in frontal or transverse plane during sagittal plane flexion–extension).

### Data analysis plan

All data were analyzed using the Statistical Package for Social Sciences (SPSS) software version 24.0 (SPSS Inc, 233 S WackerDr, 11th Fl, Chicago, IL 60606) and R Ver. 4.1.3 program. (R Foundation for Statistical Computing, Institute for Statistics and Mathematics, Welthandelsplatz 1, 1020 Vienna, Austria). The level of significance was established at p < 0.05. The distribution of the quantitative variables was tested with the Kolmogorov–Smirnov test with Lillierfors correction, which showed the absence of normality. Quantitative variables are shown as mean ± standard deviation and categorical variables with absolute and relative values (%).

Nine multivariate linear regression models were built between each of the sensorimotor control dependent variables (i.e., active maximal ROM in flexion and extension, peak velocity in flexion and extension, peak jerk in flexion and extension, peak conjunct motion in lateral and rotation movements, and head repositioning accuracy) and the secondary variables of age, previous month visual analog scale (VAS), TSK11 score, pain duration, and NDI score. The VAS score was included in the model instead of the group categorical variable (pain or asymptomatic) in order to maintain the power inherent in a continuous variable, which could be lost in a discrete categorical variable with few levels defined [43]. All patients with neck pain reported some degree of pain in the last month.

The assumption of linearity between all dependent and independent variables was tested by visual inspection of the correlation graph and a value of the effective degrees of freedom (EDF) close to 1. When this assumption was not met, a generalized additive model (GAM) was applied with the double penalty method in the selection of variables, modelling the variables with the parametric or smoothed model based on fulfilling the assumption. In all models, compliance with concurrency assumptions was tested for the smoothed terms, eliminating those with a value greater than 0.8, and multicollinearity in the parametric terms, eliminating those with a variance inflation factor (VIF) greater than 2. The distribution of residuals and adjusted values around the null value and adequacy of the number of basic functions with a non-significant K index were also checked.

In the case of the peak velocity variable, a linear model was applied when the assumptions of linearity, homoscedasticity, normality of the residuals, and absence of autocorrelation were fulfilled, while in the maximum lateral flexion a weighted least square (WLS) model was applied to be able to handle the non-normality of the residuals.

### Sample size

The sample size of this study was determined using G*Power, Version 3.1.9.2 (Franz Faul et al. University at Kiel, Germany), considering the results from a pilot study with 20 subjects: 10 asymptomatic and 10 subjects with neck pain. Sample size was calculated using a linear multiple regression (fixed model), with 0.95 power (1- beta error probability) and an alpha level of 0.05 [44]. Considering craniocervical flexion ROM as a dependent variable in a model with five independent variables, a total sample size of 160 subjects was estimated considering a partial R^2^ = 0.076 and an effect size of 0.082. Considering the probability of technical errors related to the automatic record of data from inertial sensors, an additional 20% of patients was estimated (n = 192).

## Results

Of a total of 211 participants who were screened, 19 of them were excluded due to previous surgeries in the neck and/or head region (n = 4), vestibular alterations (n = 2), otogenic or idiopathic vertigo/dizziness (n = 9), previous fracture in the head or neck region (n = 1), and osseous deformities in the thoracic, cervical, or cranial region (n = 3).

The study involved 192 participants with an average age of 34.55 ± 13.93 years (Table 1), including 124 subjects with non-traumatic neck pain and 68 asymptomatic subjects.

**Table 1:** Clinical and demographic characteristics of all participants

Variable	Neck pain (n = 124)	Asymptomatic (n = 68)
Gender n(%)		
Female	87 (70.2%)	37 (54.4%)
Male	37 (29.8%)	31 (45.6%)
Age	34.42 ± 14.1	34.6 ± 13.9
Height (cm) (Mean ± SD)	168.8 ± 9.6	165.6 ± 37.6
Weight (Mean ± SD)	66.8 ± 14.0	72.6 ± 15.8
Previous month VAS (Mean ± SD)	3.8 ± 2.3	0
Pain duration in months (Mean ± SD)	57.1 ± 80.9	0
NDI (Mean ± SD)	8.7 ± 5.4	0
TSK11 SD	19.8 ± 6.4	17.4 ± 5.9

The descriptive data of the sensorimotor control in neck pain patients and asymptomatic controls is represented in Table 2.

**Table 2:** Sensorimotor control characteristics in neck pain patients and asymptomatic controls

	Group	
	Neck pain	Asymptomatic
Craniocervical flexion ROM (degrees)	13.7 ± 11.8	14.8 ± 12.9
Craniocervical extension ROM (degrees)	3.3 ± 2.9	3.2 ± 3.8
Peak velocity in craniocervical flexion (degrees/ second)	27.7 ± 18.6	31.1 ± 18.7
Peak velocity in craniocervical extension (degrees/ second)	25.4 ± 11	28.3 ± 13.5
Peak jerk in craniocervical flexion (degrees/second^3^)	453.6 ± 247.5	565.4 ± 384
Peak jerk in craniocervical extension (degrees/second^3^)	462.5 ± 260.6	572.1 ± 373.2
Conjunct motion (rotation) (degrees)	2.2 ± 3.8	2.6 ± 3.6
Conjunct motion (lateral flexion) (degrees)	2.3 ± 2.4	2.5 ± 3.2
Head repositioning accuracy (degrees)	0.6 ± 6.8	1 ± 3

Data expressed as mean ± standard deviation or with absolute and relative values (%)

Significant explanatory variables were found in four of the models (craniocervical flexion ROM, craniocervical extension ROM, peak velocity in craniocervical flexion and lateral flexion conjunct motion). None of the remaining models found any significant explanatory variable. Table 3 shows coefficients, standard error (SE), t-values and p values for linear multivariate regression and effective degrees of freedom (EDF), reference degrees of freedom (df_ref_), F values and p values for smooth regression analysis that showed any significant explanatory variable.

**Table 3:** GAM models

Linear regression				Smooth regression			
	Coefficient (SE)	t	^a^p value		EDF (df_ref_)	F	^a^p value
*Craniocervical flexion ROM*							
Intercept	− 17.8 (2.6)	− 6.9	< 0.001	Age	5.9(7)	2	0.08
Pain duration	0.014 (0.015)	1	0.3	Previous month VAS	4.2(5.1)	1.4	0.2
NDI	0.4 (0.2)	1.3	0.2	TSK11	2.3 (3)	4.3	0.01*
*Craniocervical extension ROM*							
Intercept	1.07 (1)	1.1	0.3	TSK11	1.24 (1.442)	0.1	0.9
Age	0.1(0.02)	3.7	< 0.001*	–	–	–	–
Previous month VAS	0.1 (0.2)	0.9	0.4	–	–	–	–
Pain duration (months)	− 0.01(0.004)	− 2.4	0.02*	–	–	–	–
NDI	− 0.1 (0.1)	− 0.8	0.4	–	–	–	–
*Peak velocity in craniocervical flexion*							
Intercept	− 31.1 (4.2)	− 7.4	< 0.001	Age	6.1 (7.2)	2.4	0.03*
Previous month VAS	− 0.5 (1)	− 0.5	0.6	Pain duration	2 (2.5)	1.7	0.2
NDI	0.6 (0.4)	1.4	0.2	TSK11	8.5(9)	4.3	< 0.001*
*Conjunct motion (Lateral flexion)*							
Intercept	1 (0.4)	2	0.04	–	–	–	–
Age	0.007 (0.01)	0.8	0.4	–	–	–	–
Previous month VAS	0.1 (0.1)	0.9	0.4	–	–	–	–
Pain duration (months)	− 0.004 (0.002)	− 2.7	0.01*	–	–	–	–
NDI	− 0.1(0.03)	− 2.2	0.03				
TSK11	− 0.001 (0.02)	− 0.1	1				

*SE* standard error, *EDF* effective degrees of freedom, *df*_*ref*_ reference degrees of freedom

^*^Significant if p < 0.05

### Craniocervical flexion ROM

**Fig. 3:**
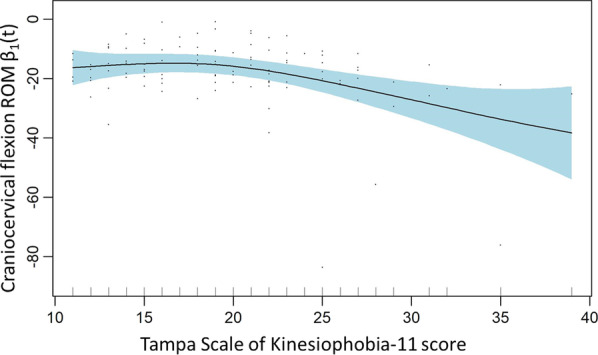
Craniocervical flexion ROM smoothed model: TSK11

A generalized additive model (GAM) for craniocervical flexion ROM using a linear regression adjusted for pain duration, and NDI and a smooth regression model adjusted for age, pain intensity (previous month), and TSK11 only showed TSK11 as a significant explanatory variable of craniocervical flexion ROM (Table 3). The effect plot (Fig. 3) shows that participants with higher scores of TSK11 have lower values of craniocervical flexion ROM. This model explained 31.8% of the variance for flexion ROM.

### Craniocervical extension ROM

A linear model showed that age and pain duration significantly explain craniocervical extension ROM (Table 3). Specifically, older subjects and those with less duration of neck pain had higher craniocervical extension ROM. This model explained 15.5% of the variance of the craniocervical extension ROM.

### Peak velocity in craniocervical flexion

**Fig. 4:**
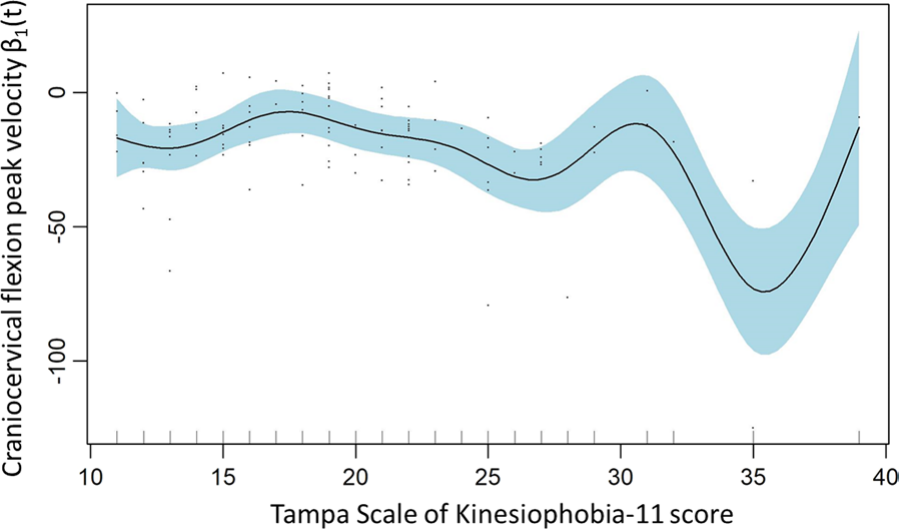
Craniocervical flexion peak velocity smoothed model: TSK11

A smoothed model showed that the variables age and TSK11 scores significantly explained the peak velocity in craniocervical flexion (Table 3). The effect plots suggest that peak velocity remains stable against age for subjects with TSK11 scores below approximately 27 points, but the few subjects with higher scores than 27 points in the TSK11 show alternate trends of association between both variables (Fig. 4). This model explained 15.5% of the variance.

### Conjunct motion (lateral flexion)

Finally, a linear model showed that pain duration and NDI significantly explained maximum conjunct motion lateral flexion (Table 3). That is, subjects with less pain duration and higher NDI scores showed increased conjunct motion. The variance explained by this model was very low (R^2^_adj_ = 0.084).

## Discussion

This study has found that sensorimotor characteristics of the craniocervical flexión movement, such as ROM, peak velocity or conjunct motion can partially be explained by the population characteristics of chronicity, age or kinesiophobia. However, the models should be interpreted with caution due to the low values of variance explained and the low absolute values of regression coefficients in the linear regression models. Therefore, the kinematic analysis of craniocervical flexion performed in this study might not be suitable to identify clear variables that could be considered relevant to explain the kinematics of the craniocervical flexion. The characteristics of the population included in this study could also have influenced our results and future research should investigate including samples with a larger proportion of neck pain patients presenting higher levels of pain, disability or kinesiophobia. Interestingly, pain intensity and self-reported disability did not partially explain any of the kinematic variables of craniocervical flexion, but other characteristics of the population, specially kinesiophobia, seemed to be more relevant factors that partially explained the kinematics features of the movement. These results agree with previous research that also observed that kinesiophobia showed stronger associations with kinematic measures of neck movement than pain intensity or disability did [45]. A recent study by Devecchi et al. [28] also observed that kinesiophobia was the most relevant factor associated with the kinematic features of patients with neck pain, supporting the fear-avoidance model, which considers fear of motion as a potential driver for physical deconditioning and a mediator between pain and disability in people with neck pain [46].

### Craniocervical flexion range of motion

Maximum ROM in craniocervical flexion was only significantly explained by kinesiophobia. In this smoothed model, higher scores in the TSK11 were associated with lower values of ROM (Fig. 3). This trend seemed to occur only for high scores of TSK11 superior to approximately 20 points (Fig. 3). This is the model that showed the highest values of explained variance in our study (31.8%). Although the explained variance is limited, it should be considered that we observed a great amount of inherent variation in sensorimotor control values between individuals with similar characteristics, including maximal craniocervical flexion ROM (Table 1), which may be associated with the low percentages of explained variance in the regression models [47].

It can be hypothesized that patients in our study with higher fear of movement may perceive the final degrees of craniocervical flexion as a more threatening demand due to the feeling of reaching the end of the ROM, usually perceiving the sensation of tension of the cervical myodural bridge [48] or stretch of structures of the neck. However, the results on this relationship should be interpreted with caution and this possible association should be further investigated in large samples of patients with higher levels of kinesiophobia.

To the authors’ knowledge, the possible association between kinesiophobia and reduced ROM in craniocervical flexion have been scarcely investigated, and there has not been an association observed between these variables [49]. Studies investigating other global cervical movements have showed variable results, since kinesiophobia seemed to influence the ROM during task-oriented movements [45], but did not associate with flexion–extension ROM during active planar movements [50].

Other factors were investigated in this model, including the intensity or chronicity of pain, age, or the level of disability, but these did not associate with the maximal ROM in craniocervical flexion. To the authors’ knowledge, there is sparse research that has investigated the influence of pain, disability, or psychological variables on the maximal active ROM during the craniocervical flexion movement specifically, nor on any other of the sensorimotor control variables analyzed in this study. In agreement with our results, a study by Rudolfsson et al. [49] did not observe an evident difference between neck pain patients and healthy controls on the maximum ROM in craniocervical flexion. Moreover, a study by Ernst et al. [29] did not show a clear correlation between neck pain intensity and craniocervical flexion ROM in a sample of neck pain patients. However, it should be noted that our study could not be directly compared with these studies, since the measuring position, the measuring instrument, the instructions provided to participants, and/or the values of ROM reported are different compared to our study. Moreover, out study considered pain and disability levels as continuous variables and not as categories of the sample.

Results from previous studies in the analysis of full cervical active ROM during planar movements (flexion–extension, rotations and lateral flexion) have showed good quality of evidence that there is a significantly, clinically relevant decrease in cervical ROM in patients with non-traumatic neck pain when compared to asymptomatic controls [15]. In our study, an influence of pain on active craniocervical flexion ROM was not observed, suggesting that the specific assessment of craniocervical flexion active ROM may not be a clinically relevant parameter when evaluating patients with neck pain. However, the levels of pain and disability of our study population were low or moderate in most cases, which limits the generalizability of our results.

The method used in our study to guide participants in order to perform the craniocervical flexion movement is based on several aspects considered when performing the CCFT, in terms of the patient position or the set of verbal instructions provided. A systematic review and meta-analysis by Romeo et al. [30] recently reported that the CCFT has shown a good ability to discriminate between individuals who are asymptomatic and individuals with neck pain. It should be noted that the sensorimotor demands assessed by the CCFT may be different compared to the performance of the full-range active craniocervical flexion evaluated in the present study. The CCFT demands the performance and maintenance of progressive activations of DCF in time based on a set of stages with high precision and increasing load, while in our study participants performed three repetitions of a maximal craniocervical flexion, which were maintained for a few seconds, likely a different and less demanding task for the sensorimotor system. Future research should investigate the kinematics of craniocervical flexion during the more demanding versions of the CCFT.

### Craniocervical extension ROM

The analysis of the craniocervical extension ROM in this study does not represent the maximal craniocervical extension participants can reach in an independent motion analysis, but rather the maximal ROM reached when the participant is trying to return to the neutral position from the craniocervical flexion motion. Therefore, we hypothesize that higher values in this variable could be considered as a lack of control when trying to progressively come from flexion to extension when trying to reach the neutral position. Most of the patients performed few degrees of craniocervical extension when returning to the neutral position and then progressively performed a small craniocervical flexion motion to try to more accurately reach the neutral position.

The results of this linear model showed that increased age and lower chronicity of pain were significant explanatory variables of higher values of craniocervical extension. However, these results are limited by the low explained variance (15.5%) of the model. Moreover, when looking at the regression coefficients, described as the change in the dependent variable for each one-unit change in the independent variable [51], the observed values of ROM do not seem to be clinically meaningful for pain chronicity measured in months (coefficient = − 0.009). Therefore, only age (coefficient = 0.081) could have some consideration as a explanatory variable. It can be hypothesized that older patients could have reduced sensorimotor control when trying to return to the neutral position. However, the low values of explained variance of the model require further research with older populations to draw definitive conclusions.

To the authors’ knowledge, no previous research has investigated the factors that can influence this specific craniocervical extension ROM when returning from maximal craniocervical flexion.

### Velocity and jerk

**Fig. 5:**
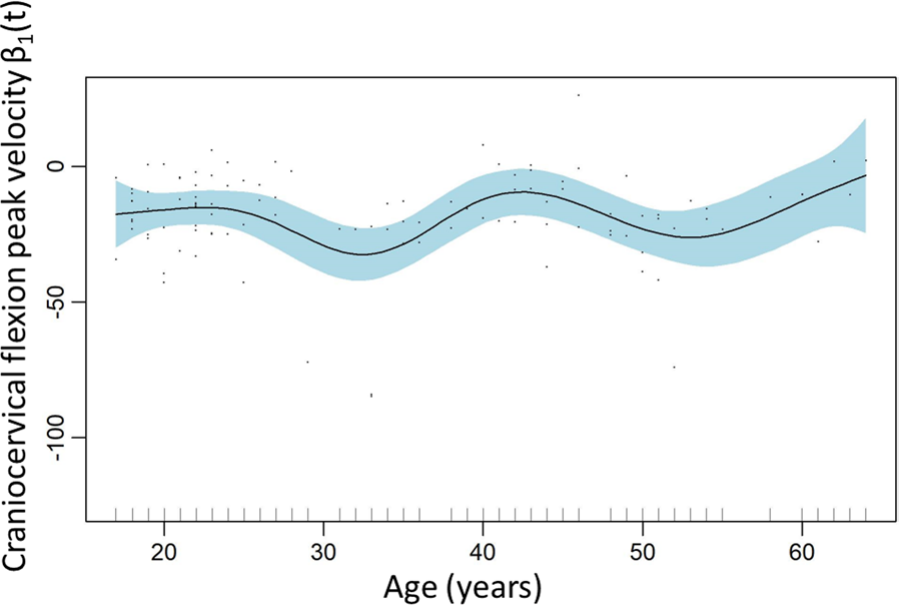
Craniocervical flexion peak velocity smoothed model: Age

Velocity and jerk were evaluated both during the movement from neutral to craniocervical flexion (flexion movement) and during the maximal craniocervical flexion to the neutral (extension movement). Only the smoothed model for velocity during craniocervical flexion showed the significant explanatory variables of age (p = 0.028) and kinesiophobia (p < 0.001). However, these results could only explain 15.5% of the variance. Moreover, the smoothed terms partial effect plot for age do not show a clear trend for its interpretation, since it remains stable with alternative small variations across different ages (Fig. 5). The plot for kinesiophobia (Fig. 4) shows a decreasing tendency from scores of 30 to 35 and an inversion of this tendency from scores of 35 to 40, but the relevance of these observations may not be meaningful because there are only a few cases in the sample with scores above 30 or 35 in the TSK11.

These results of this study suggest that the specific assessment of craniocervical flexion velocity or jerk may not be a clinically relevant parameter to assess in patients with neck pain. Future research is needed to further confirm this perception in a population with higher levels of pain or disability. To our knowledge, no previous research has investigated the factors that can explain velocity or jerk variables during the craniocervical flexion movement. However, there is some evidence of alterations of movement velocity or jerk during other global cervical planar or functional movements in patients with mechanical neck pain compared to asymptomatic controls [16–18].

### Conjunct motion

Conjunct motion was analyzed as the maximal ROM achieved in any other plane that is not the flexion–extension sagittal plane during the craniocervical flexion. This variable has been investigated in previous research during other movements and considered as an alteration in sensorimotor control that might reflect protective or learned strategies [41, 50, 52]. None of the independent variables explained conjunct motion in the rotation plane. Pain duration and neck disability significantly explained less conjunct motion in lateral flexion. However, the explained variance of the model is very low (R^2^_adj_ = 0.084), which limits the relevance of these findings. Previous studies have not observed increased conjunct motion in patients with neck pain when assessed during other global cervical movements, or have observed reduced conjunct motion in patients with neck pain [16]. Similar to other variables analyzed in previous models, the results of our study do not support the assessment of conjunct motion during maximal craniocervical flexion movement in patients with neck pain. It can hypothesized that the low-range guided craniocervical flexion motion analyzed in our study might not facilitate the occurrence of large conjunct motion when compared to other wide range global movements performed freely and unsupported. However, future research could investigate this variable in larger samples of neck pain patients with high pain or disability levels.

### Head reposition accuracy

None of the independent variables significantly explained the joint position error when patients returned to the neutral starting position. Head repositioning accuracy during the performance of maximal craniocervical flexion divided in three independent repetitions may not be a clinically relevant variable to assess in patients with neck pain. However, future research is needed to further investigate its influence in large samples of patients with high levels of pain,disability and kinesiophobia. Previous systematic reviews evaluating head repositioning accuracy when returning to a neutral head position following planar movements of rotation or flexion–extension have suggested differences between idiopathic neck pain and healthy groups [10, 14]. However, the clinical relevance of these differences was considered questionable, showing the need of further research on the clinical meaning of statistically significant differences [14].

### Limitations

To our knowledge, this is the first study to investigate the use of a novel inertial technology to assess further sensorimotor control characteristics during the performance of the craniocervical flexion movement. However, the present study has various limitations. First, associations across variables were investigated through a cross-sectional design, so no inferences can be made in terms of causality. Future data from longitudinal studies could provide more evidence on the possible causal direction of the relationship between variables. Second, the study population characteristics may be limited to young individuals of European descent with low to moderate levels of pain, disability, or kinesiophobia. Future research could investigate larger samples with high levels of these variables. Third, although the instructions to perform the craniocervical flexion movement were standardized, subjects of older age or with a lower educational level may present more difficulties in understanding the correct performance of the movement. Fourth, the muscle activation of deep and superficial muscles while performing the craniocervical flexion was not monitored by electromyography. It is possible that patients could have changed their motor strategies to achieve similar kinematic performance during the movement. Previous research has shown the complexity and variability of neuromuscular adaptations in patients with pain [53]. Fourth, our study did not analyze the sensorimotor control during the performance of the CCFT, but only during three repetitions of the full-range craniocervical flexion movement. Future research could use inertial sensor technology to further investigate the kinematics during the performance of the original CCFT.

## Conclusion

The results of this study did not show a clear relationship between the population characteristics and the sensorimotor control performance. Therefore, the kinematic analysis of craniocervical flexion performed might not be suitable to identify clear variables that could be considered to relevantly explain the kinematics of the craniocervical flexion. Kinesiophobia might show some degree of association with reduced ROM, but further research in large samples of patients with higher levels of chronic pain, disability and kinesiophobia is needed.

## Data Availability

The datasets generated during the current study are available from the corresponding author upon request.

## Abbreviations

CCFT: Craniocervical flexion test
EDF: Effective degrees of freedom
GAM: Generalized additive model
NDI: Neck disability index
ROM: Range of motion
TSK11: Tampa scale of kinesiophobia-11
VAS: Visual analog scale
